# Integrated Polygenic Tool Substantially Enhances Coronary Artery Disease Prediction

**DOI:** 10.1161/CIRCGEN.120.003304

**Published:** 2021-03-02

**Authors:** Fernando Riveros-Mckay, Michael E. Weale, Rachel Moore, Saskia Selzam, Eva Krapohl, R. Michael Sivley, William A. Tarran, Peter Sørensen, Alexander S. Lachapelle, Jonathan A. Griffiths, Ayden Saffari, John Deanfield, Chris C.A. Spencer, Julia Hippisley-Cox, David J. Hunter, Jack W. O’Sullivan, Euan A. Ashley, Vincent Plagnol, Peter Donnelly

**Affiliations:** 1 Genomics plc, Oxford, United Kingdom (F.R.-M., M.E.W., R.M., S.S., E.K., R.M.S., W.A.T., P.S., A.S.L., J.A.G., A.S., C.C.A.S., V.P., P.D.).; 2 Institute of Cardiovascular Sciences, University College London, United Kingdom (J.D.).; 3 Department of Primary Care Health Sciences (J.H.-C.), University of Oxford, United Kingdom.; 4 Nuffield Department of Population Health (D.J.H.), University of Oxford, United Kingdom.; 5 Division of Cardiology, Department of Medicine, Stanford University School of Medicine, CA (J.W.O., E.A.A.).

**Keywords:** coronary artery disease, genetic epidemiology, genetics, primary prevention, risk factors

## Abstract

**Background:**

There is considerable interest in whether genetic data can be used to improve standard cardiovascular disease risk calculators, as the latter are routinely used in clinical practice to manage preventative treatment.

**Methods:**

Using the UK Biobank resource, we developed our own polygenic risk score for coronary artery disease (CAD). We used an additional 60 000 UK Biobank individuals to develop an integrated risk tool (IRT) that combined our polygenic risk score with established risk tools (either the American Heart Association/American College of Cardiology pooled cohort equations [PCE] or UK QRISK3), and we tested our IRT in an additional, independent set of 186 451 UK Biobank individuals.

**Results:**

The novel CAD polygenic risk score shows superior predictive power for CAD events, compared with other published polygenic risk scores, and is largely uncorrelated with PCE and QRISK3. When combined with PCE into an IRT, it has superior predictive accuracy. Overall, 10.4% of incident CAD cases were misclassified as low risk by PCE and correctly classified as high risk by the IRT, compared with 4.4% misclassified by the IRT and correctly classified by PCE. The overall net reclassification improvement for the IRT was 5.9% (95% CI, 4.7–7.0). When individuals were stratified into age-by-sex subgroups, the improvement was larger for all subgroups (range, 8.3%–15.4%), with the best performance in 40- to 54-year-old men (15.4% [95% CI, 11.6–19.3]). Comparable results were found using a different risk tool (QRISK3) and also a broader definition of cardiovascular disease. Use of the IRT is estimated to avoid up to 12 000 deaths in the United States over a 5-year period.

**Conclusions:**

An IRT that includes polygenic risk outperforms current risk stratification tools and offers greater opportunity for early interventions. Given the plummeting costs of genetic tests, future iterations of CAD risk tools would be enhanced with the addition of a person’s polygenic risk.

## Abstract 2

Supplemental Digital Content is available in the text.

Cardiovascular disease (CVD) is a major cause of morbidity and mortality worldwide.^1^ A risk-based prevention strategy, with prevention efforts most strongly targeted toward those at higher risk, is the widely accepted approach to disease prevention.^2^ In the United States, the risk prediction algorithm recommended by the American College of Cardiology (ACC)/American Heart Association (AHA) is the pooled cohort equations (PCE) algorithm,^3,4^ while in the United Kingdom, the National Institute for Health and Care Excellence recommends the QRISK algorithm, with the latest version being QRISK3.^5,6^ Both algorithms predict CVD risk over 10 years, based on multiple risk factors including age, sex, ethnicity, smoking history, systolic blood pressure, cholesterol levels, and comorbidities. Active management, which may include lipid-lowering treatment, is recommended for individuals whose 10-year risk is predicted to be above a certain threshold (7.5% in the United States and 10% in the United Kingdom).

An important component of CVD is coronary artery disease (CAD), which has been a particular focus for genetic studies. Family and genome-wide association studies (GWAS) have estimated a heritability for CAD of between 40% and 60%.^7,8^ More recently, studies have shown that a polygenic risk score (PRS) derived from large-scale genome-wide genotype data can have predictive power for CAD,^9–11^ raising the question of whether it would be beneficial to add PRS to existing risk predictors to aid the identification of high-risk individuals.^12–15^ As it remains constant over the life course, a PRS could be used to guide disease prevention earlier in life before standard risk factors have an appreciable impact.

Three recent studies in middle-aged individuals of European ancestries have combined a PRS for CAD with standard risk prediction algorithms.^16–18^ One large study of 352 660 adults in the UK Biobank (UKB) found a significant overall net reclassification improvement (NRI; 4.4% against the PCE algorithm [95% CI, 3.1–4.9])^16^ and discrimination when stratifying by age (≥55 and <55 years old). Smaller studies in the ARIC cohort (Atherosclerosis Risk in Communities) (n=4847),^17^ MESA cohort (Multi-Ethnic Study of Atherosclerois) (n=2390),^17^ and the FINRISK cohort (n=21 813)^18^ found a nonsignificant NRI for risk tools that combined PRS and standard risk tools, compared with the standard risk tool alone. The FINRISK study (Finland Risk) also examined predictive performance in ≥55- and <55-year-old age groups and found a significant NRI in the younger group but not in the older group.

While some of the above studies examined the predictive accuracy of an integrated PRS and clinical risk tool in different age groups, this stratification was limited (only <55 or ≥55 years old) and did not additionally stratify age groups by sex. Given the uncertainty surrounding the clinical utility of an integrated genetic and clinical cardiovascular risk tool, we set out to definitively address this. With our access to the largest GWAS results and enhanced methods to construct PRS (using a combination of novel and established methodologies), we examine the clinical utility of an integrated genetic and clinical risk prediction tool both overall and across a broad array of age-by-sex subgroups.

## Methods

The detailed methods of this work are available in the Data Supplement. All UKB individuals have given informed consent. Our research project (project application number 9659) was approved by the UKB according to their established access procedures,^19^ and legal and ethical approval is covered by the Research Tissue Bank approval obtained from the UKB’s governing Research Ethics Committee (REC 16/NW/0274), as recommended by the National Research Ethics Service. To facilitate reproducibility of our results while also respecting the sensitive individual-level nature of these data, the values of the PRSs using our novel CAD PRS for the 186 451 individuals on whom they were evaluated in this study, along with relevant case-control indicators, will be returned to UKB so that they can be made available to approved UKB researchers. Additionally, the UKB group I GWAS summary statistics can be accessed at https://zenodo.org/record/4421038.

## Results

### CAD PRS Performance

**Figure 1.**
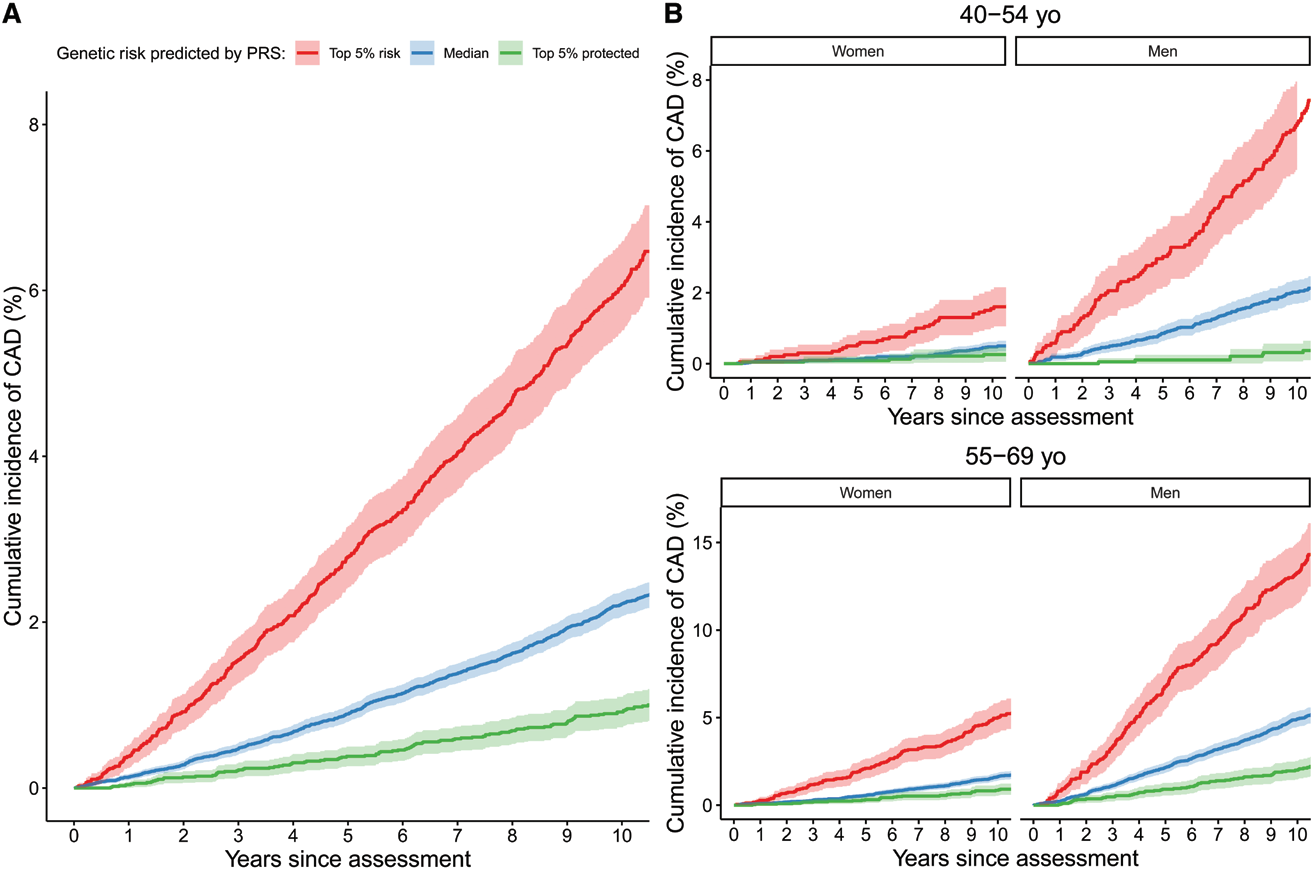
**Cumulative incidence of coronary artery disease (CAD) in UK Biobank incident cases in group III.** **A**, All of group III. **B**, Group III stratified into 4 subgroups according to age (45–54- and 55–69-y-old age ranges) and sex. Individuals are further stratified by polygenic risk score (PRS)–defined risk into the top 5% of PRS risk (red), the median 40% to 60% distribution of risk (blue), and the bottom 5% of risk distribution (green).

We considered first the independent predictive performance of the CAD PRS, both overall and separated into age-by-sex subgroups. Overall, our PRS was a significant predictor of CAD in both prevalent (pre-UKB assessment) and incident (post-UKB assessment) outcomes (prevalent nonevents and events obtained from groups III and IV, respectively: Harrell’s C, 0.69; hazard ratio per SD increase, 1.90 [95% CI, 1.86–1.95]; *P*<10^100^; incident outcomes obtained from group III: Harrell C, 0.63; hazard ratio per SD increase, 1.62 [95% CI, 1.57–1.67]; *P*<10^100^; Figure 1A). A similar pattern of separation by PRS-defined risk in the cumulative incidence of CAD was also seen in all age-by-sex subgroups (Figure 1B), with the pattern seen most clearly in men due to their greater incidence of CAD events.

We compared the performance of our PRS against that recently derived and used by Elliott et al,^16^ the earlier PRS of Khera et al^10^ (which was used in the analysis of Mosley et al^17^), and also the PRS derived by Inouye et al.^9^ We were unable to conduct a formal comparison in UKB against the PRS developed by Mars et al,^18^ as they used the entirety of UKB to construct their PRS. Published weights for the other 3 PRSs were downloaded and reapplied within our pipeline, to provide a like-for-like comparison. Our PRS is the most powerful, followed by the Inouye et al and Khera et al PRSs (with similar performances) and then the Elliott et al PRS (Table 1). Focusing on incident outcomes (group III), the Harrell C statistic of our PRS was higher than the second best-performing PRS (Inouye et al, *z* test *P*=4.6×10^−6^ for a Harrell C difference of 0.015).

**Table 1.**
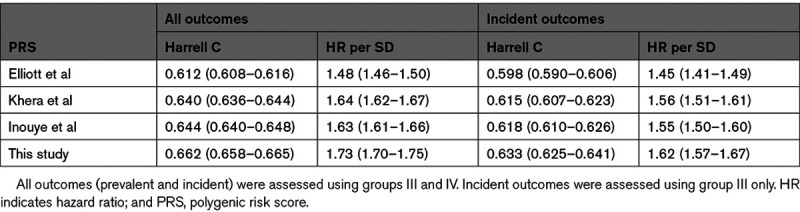
Prediction Performance Metrics (With 95% CI) of Our PRS Compared With That Used by Elliott et al,^16^ Khera et al,^10^ and Inouye et al^9^

We used generalized survival analysis to examine whether the performance of our PRS varied significantly by age. We found evidence in men, but not in women, that predictive power is the highest at younger age groups and declines for older ages (Figure I in the Data Supplement). In contrast, PRS performance (as measured by hazard ratio per 1 SD increase) did not significantly differ between men and women (interaction test *P*=0.2 from survival analysis).

We compared the predictive accuracy of our CAD PRS to the individual effects of other known risk factors for CAD. Measured via Harrell C, the CAD PRS has similar predictive power to each of systolic blood pressure, HDL (high-density lipoprotein) cholesterol, and LDL (low-density lipoprotein) cholesterol and is more powerful than either total cholesterol or smoking history (Figure II in the Data Supplement). The C values vary by age for many of the factors. The only significant difference by sex within age group (at *P*=6×10^−3^ after Bonferroni correction for multiple comparisons) is for PRS in the younger (40–55 years old) age group. The CAD PRS in 40- to 55-year-old men was more predictive than any other single risk factor in any other group.

### Integrated Risk Tool Performance

We investigated PCE applied to CAD outcomes as the basis for our primary analysis (see Tables I–IV in the Data Supplement and Figure III in the Data Supplement for secondary analyses relating to a second risk predictor, namely QRISK3, and to a second disease outcome, namely CVD, with qualitatively similar, but less strong, results, and see Table V in the Data Supplement for an analysis using additional criteria to define CVD-free individuals, with similar results). We first checked that PCE was a strong predictor in its own right, as previous work had suggested that if PCE is a poor predictor, then it becomes an easily beaten straw man.^20^ Our overall Harrell’s C for PCE was 0.76 (95% CI, 0.75–0.76), comparable to that reported by Elliott et al^16^ (overall C, 0.76 [95% CI, 0.75–0.77]), and generally reflective of good prediction.^20^

We proceeded to assess whether CAD PRS further enhanced risk prediction beyond the PCE predictions. We first calculated the correlation between an individual’s PCE score (log-odds scale) and their CAD PRS and found that these were largely uncorrelated (Pearson correlation coefficient *r*, 0.016; a similar result was found for QRISK3 *r*, 0.025). We note that the two largest contributors to PCE scores are age and sex, which under standard conditions are uncorrelated with PRS. Family history of premature CVD is partly a proxy for genetic risk and is used as a risk factor in QRISK3 and alongside PCE for risk management in the United States.^21^ We found that our PRS was largely uncorrelated with first-degree-relative family history of CVD in UKB (*r*, 0.084), but we note that age of event is not captured in UKB, so we were not able to establish the correlation of our PRS with a family history of premature CVD.

**Figure 2.**
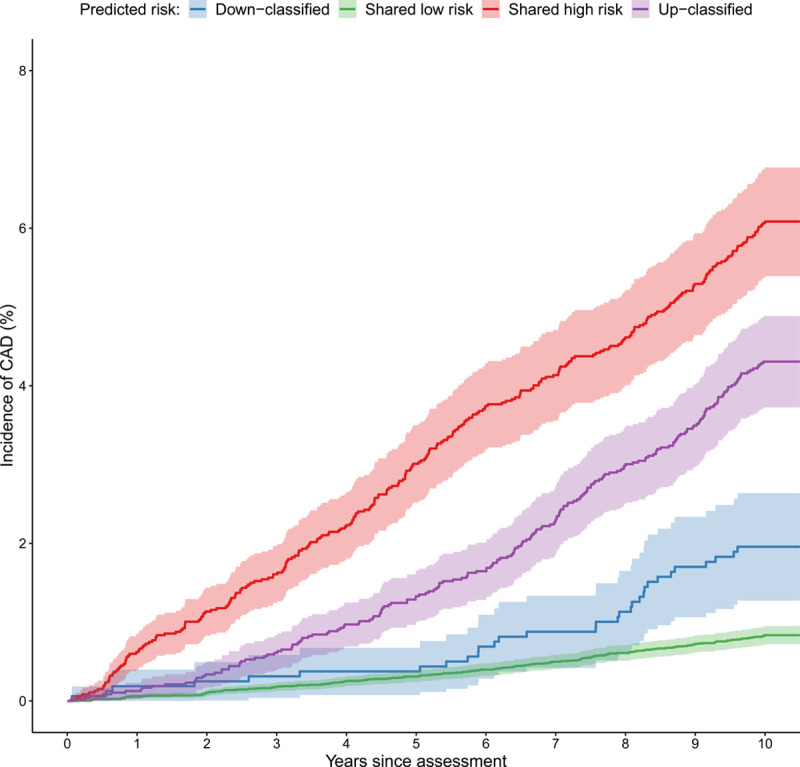
**Cumulative incidence of coronary artery disease (CAD) in the subgroup of 40- to 54-y-old men in group III.** Individuals are stratified by pooled cohort equations (PCE) and integrated risk tool (IRT)–defined risk (above/below the 7.5% threshold) into those predicted to be high risk by both PCE and IRT (red), those up-classified to high risk by IRT (purple), those down-classified to low risk by IRT (blue), and those predicted to be at low risk by both PCE and IRT (green).

We then investigated reclassification patterns, comparing our integrated risk tool (IRT) with PCE and using a 7.5% 10-year risk threshold to define high- and low-risk groups as recommended under the ACC/AHA guidelines^4^ (Table 2). We found substantial reclassification movements between the PCE and IRT models. Overall, 13.7% of individuals are reclassified, of which 7.0% are reclassified from low to high risk and 6.7% are reclassified from high to low risk by the IRT. There are also substantial differences by age and sex in the overall number of people that are reclassified by the IRT model, with the overall rate peaking in men at 50 to 54 years of age and in women at 65 to 69 years of age (Figure IV in the Data Supplement). Evidence that these reclassifications are beneficial is indicated by the observation that 10.4% of incident cases are correctly up-classified by the IRT, compared with 4.4% that are incorrectly down-classified. Further evidence for beneficial reclassification is provided by a comparison of cumulative CAD incidence in different reclassification groups, which shows that individuals who were up-classified by our IRT had consistently greater cumulative CAD incidence than those down-classified by our IRT (Figure 2). This pattern is also seen to varying degrees in the age-by-sex subgroups (Figure V in the Data Supplement).

**Table 2.**
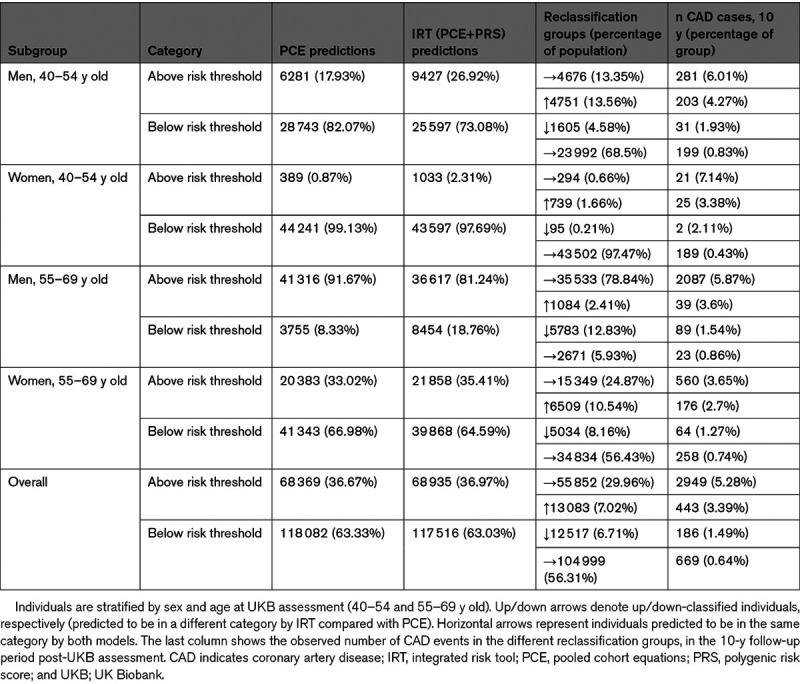
Reclassification Numbers for Our IRT (PCE+PRS) Model Compared With PCE Alone in Group III

**Figure 3.**
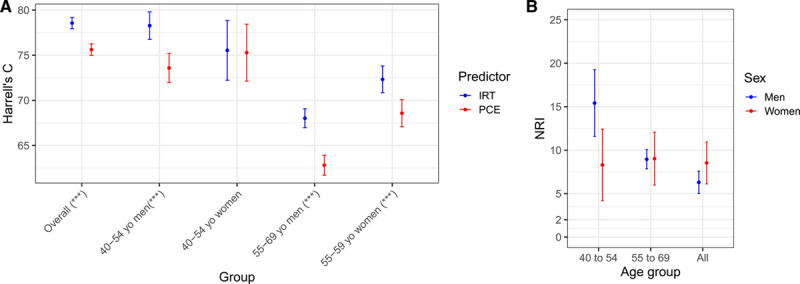
**Model discrimination and net reclassification improvement for the integrated risk tool (IRT) compared with pooled cohort equations (PCE).** **A**, Harrell’s C overall and across age-by-sex subgroups. Blue and red lines refer to IRT and PCE, respectively. Asterisks in *x* axis labels denote level of significance for the difference in Harrel C (***P<0.001). **B**, Net reclassification improvement (NRI) for the IRT compared with PCE alone across different age groups in men (blue) and women (red). The bars indicate the 95% CIs.

Next, we analyzed differences in model discrimination and NRI (Table 3; Figure 3). Overall, the difference in Harrell C was 3% (95% CI, 2%–4%) and ranged from 0% to 5% in the age-by-sex subgroups. The overall NRI was 5.9% (95% CI, 4.7%–7.0%). The positive changes in full NRI were all strongly significant even after Bonferroni correction for multiple testing (maximum *P*=0.005 after correcting for the overall test plus 4 age-by-sex tests), while the difference in Harrell C was significant overall (corrected *P*=7.7×10^−12^), in the two male subgroups (men 40–54 years old corrected *P*=1.0×10^−4^; men 55–69 years old corrected *P*=4.4×10^−9^) and in the older female subgroup but not in the younger female subgroup (women 55–69 years old corrected *P*=5.4×10^−3^). Stated in traditional diagnostic terminology (sensitivity, specificity, and positive predictive value) at the established 7.5% 10-year risk threshold, our rescaling approach holds the positive predictive value constant while improving sensitivity at a moderate specificity cost (Table VI in the Data Supplement). The parameters of this trade-off vary depending on the subset of the population being considered (Table VI in the Data Supplement).

**Table 3.**
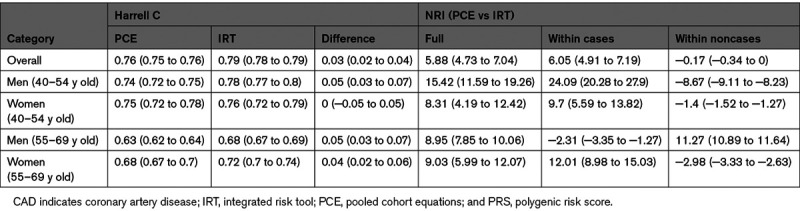
Prediction Performance Metrics (With 95% CI) for Incident CAD Outcomes in Group III, Comparing PCE and IRT (PCE+PRS) Models and Stratifying Into Age-by-Sex Subgroups

When broken down into age-by-sex subgroups, we observed that all the subgroup NRIs were larger than the overall NRI, ranging from 8.3% to 15.4% and with the largest improvement seen in younger middle-aged men (40–54 years old; Table 3). This behavior is driven by different types of positive reclassification in younger (40–54 years old) versus older (55–69 years old) middle-aged men, which to some extent cancel out in the overall NRI. In younger men, the large positive NRI (15.4% [95% CI, 11.6%–19.3%]) is driven by a large NRI in cases (24.1% [95% CI, 20.3%–27.9%]), while in older men, the positive NRI (9.0% [95% CI, 7.9%–10.1%]) is driven by a positive NRI in noncases (11.3% [95% CI, 10.9%–11.6%]; Figure 3B; Figure VI in the Data Supplement).

Finally, we compared the performance of our IRT with an alternative algorithm for combining PRS with PCE,^22^ in which individuals at borderline risk (5%<PCE<7.5%) are promoted to actionable risk if their PRS is high enough to be considered a risk-enhancing factor (in the top 20% of the PRS distribution). We found our IRT to be superior to this alternative algorithm based on combined NRI metrics, both overall and when split into age-by-sex subgroups (Table VII in the Data Supplement).

### Estimating the Overall Number of Lives Saved

Using an approach described in the Data Supplement that leverages previous work by Yang et al,^23^ we predict that 2423 (≥95% CI, 560–4059) deaths would be preventable annually in the United States from our IRT under 100% statin therapy uptake and compliance, while 1551 (≥95% CI, 358–2598) deaths would be preventable under 64% uptake and compliance.

## Discussion

In our study, we developed a new PRS for CAD and then considered its predictive performance both on its own and when used as part of an IRT by combining the CAD PRS with established clinical risk prediction tools based on nongenetic risk factors (PCE and QRISK3 as recommended in the United States and United Kingdom, respectively). We evaluated the performance of the IRT in a large independent test set of 186 451 participants in the UKB, both overall and in participants stratified into age-by-sex subgroups.

In contrast to previous studies,^16–18^ we found that our IRT performed substantially better than the established risk prediction tools. Previous studies reported overall NRIs in the range 0.1% to 4.4%. We report an overall NRI of 5.9% compared with PCE. This numerical increase translates to substantive clinical utility, as we discuss below.

There are several ways in which our study differs from the other 3 cited above, starting with the cohorts investigated. We matched our definition of CAD and CVD to that of the previous study also performed in UKB,^16^ but our studies, nevertheless, differ in that we took advantage of a more recent release of incident data. We recognize, therefore, that multiple factors might explain the differences in predictive performance, but we note that our own PRS for CAD, which combined the largest available published CAD GWAS with additional UKB data, provides more powerful prediction on its own than the PRS published by Khera et al^10^ (used by Mosley et al^17^) and by Inouye et al^9^ and is also substantially more powerful than the PRS developed by Elliott et al.^16^ We also found our CAD PRS to be as powerful for prediction as each of several established measured risk factors (systolic blood pressure, HDL, and LDL cholesterol) and more powerful than others (eg, total cholesterol and smoking history). It is the also the best-performing single risk factor in younger middle-aged men (40–54 years old).

Additionally, we analyzed in detail the breakdown of reclassification losses and gains making up the NRI. Net classification improvements were found within all age-by-sex subgroups at a level higher than the overall figure (ranging from 8.3% to 15.4%). A particularly striking value of 15.4% was seen for younger middle-aged men (40–54 years old). A net additional 24.1% of cases are identified by our IRT in this subgroup that are overlooked by the PCE tool. While further studies will be needed to fully explain these patterns, we speculate that 2 processes may contribute. One is that earlier CVD events may be more genetically determined than older ones. For example, a recent study found that 17.3% of patients aged <55 years with hospitalized early-onset myocardial infarction possessed a CAD PRS of equivalent risk to a familial hypercholesterolemia mutation.^24^ In parallel, the PCE algorithm is trained on CVD events that are most numerous in older age groups, and one consequence of this is that the risk factors it uses tend to develop only later in life.

Regardless of underlying mechanisms, it is important to note that all subgroups appear to benefit from a PRS-based IRT, so we do not propose that a PRS-based IRT is applied only to younger middle-aged men. Nevertheless, an advantage of PRS as a risk factor is that it can also be used earlier in an individual’s life to identify those who may have high lifetime CAD risk but before most other nongenetic risk factors have developed predictive power. We have also shown that the new CAD PRS is largely uncorrelated with PCE and QRISK3, which underlines its utility as an independent risk factor.

There are limitations to our study, most of which are shared by other PRS studies. Our study was performed in the UKB and is, therefore, limited by the characteristics of this cohort and by the lack of additional external datasets for evaluation. In particular, the cohort is of primarily European ancestries (and was restricted to European ancestries in this study), the age range of participants at UKB assessment is limited to 40 to 69 years, and participants tend to be healthier and more affluent than the general UK population.^25^ Additional limitations include the assessment of a simple single-risk-assessment-at-baseline scenario rather than a continuous-assessment scenario, a blanket exclusion of samples with missing data for training and testing, a PRS that is constructed from common variants without rare high-risk variants, an evaluation of PCE and QRISK3 risk tools only, a reliance on UKB data that included self-report to define some outcomes and variables, and the use of prevalent CAD cases in the construction of the PRS.

An additional limitation of our study, which is common to all PRS studies so far, is that we define a single PRS and apply it uniformly to all individuals, regardless of their age, sex, or other relevant factors. This approach assumes that, throughout the genome, common variant genetic effects are independent of these other factors. In contrast, we have shown here that PRS prediction varies by age in men. Furthermore, the attenuated effect of PRS in individuals in non-European ancestries resulting from biased study data collection is well documented.^26^ It is possible, although currently unproven, that the genetic determinants of a trait like CAD may vary across other population strata (defined, for example, by age, sex, or socioeconomic background). If so, we note that constructing group-specific PRSs may further improve predictive power if there were sufficient data for training.^27^ Even without these more sophisticated statistical approaches, the predictive power of PRSs will also increase as more population-scale data become available, especially in individuals of non-European ancestries.

One potential concern with using PRS to identify high-risk individuals is whether existing interventions are effective in genetically defined risk groups. Studies performed so far are reassuring in this regard. Statin therapy^28,29^ and PCSK9 inhibition^30^ is at least as effective in individuals with high CAD PRS and may in fact be more effective than average. Lifestyle changes involving diet and exercise are also effective in this group.^31^

Our results have a number of potential clinical implications. The ACC/AHA currently recommends considering a statin prescription if one’s 10-year risk of an atherosclerotic CVD is ≥7.5%.^4^ Currently, this risk is determined entirely from clinical risk factors. Our results indicate that risks are more accurately predicted if one’s PRS is included. Thus, some patients who are classified as at <7.5% risk using the current ACC/AHA risk score will be at ≥7.5% risk when their PRS is incorporated. This means, using the current risk score, that many patients are not offered statin therapy when they do in fact have a ≥7.5% risk. The improvements in classification are even more pronounced among age and sex subgroups (Table 3).

Cardiovascular risk tools have continually evolved as additional risk factors have been shown to improve a tool’s predictive ability, for example, with the addition of diabetes status, which was not part of the original Framingham risk score.^3,32^ Our results show enhanced predictive ability when PRS is incorporated into the current ACC/AHA atherosclerotic CVD risk score (PCE), and we show that we can substantially improve the classification of a patient’s 10-year risk. It, therefore, appears that future iterations of the ACC/AHA score (and other equivalent scores) might be more accurate if PRS was included as an additional risk factor, just as the ACC/AHA risk model was improved with the addition of diabetes status.

In the United States, the CVD risk assessment is recommended in people aged 40 to 75 years,^4^ of whom ≈105 million are atherosclerotic CVD free at any one time.^23^ The addition of our PRS to PCE would up-classify 7% of the population to a level of cardiovascular risk that warrants statin prevention (≈7.4 million individuals), which we estimate could lead to ≈12 000 lives saved over 5 years. We believe this creates a motivation for the incorporation of PRS into clinical practice, although additional feasibility studies are still required. As an alternative to our IRT approach, a previous study^22^ proposed that high genetic risk for CAD could be included as a risk-enhancing factor for individuals with borderline PCE score (between 5% and 7.5%), and this proposal fits well with existing US blood cholesterol management guidelines.^21^ Our IRT outperforms this approach and has the advantage of potentially up-classifying more individuals (including individuals with PCE scores below 5% but with high PRS), as well as down-classifying some of the individuals with PCE scores above 7.5%.

We, therefore, conclude that the addition of the PRS enhances the predictive ability and clinical utility of existing CAD risk tools. This enhanced predictive ability is especially pronounced in younger middle-aged men (40–54 years old), but it is seen across all studied age groups and across sexes. Our study argues that future iterations of PCE (and similar tools) may benefit from the addition of PRSs.

## Acknowledgments

We thank all the participants and scientists who generated and shared the data from the UK Biobank and other data sources that were used in this study. GWAS data on coronary artery disease have been contributed by CARDIoGRAMplusC4D investigators and have been downloaded from www.cardiogramplusc4d.org. The use of UK Biobank data has been conducted under project application number 9659. Drs Plagnol and Donnelly jointly supervised this study.

## Sources of Funding

This study was funded by Genomics plc.

## Disclosures

Drs Riveros-Mckay, Weale, Moore, Selzam, Krapohl, Sivley, Tarran, Sørensen, Lachapelle, Griffiths, Saffari, Spencer, Plagnol, and Donnelly are employees, and Dr Donnelly is a director of Genomics plc—a genomics health care company with an interest in the application of genetics to precision health. Dr Donnelly is also a partner in Peptide Groove LLP. The other authors report no conflicts.

## Supplemental Materials

Online Tables I–X

Online Figures I–VII

References^23–32^
